# Distinct dynamics of mRNA LNPs in mice and nonhuman primates revealed by in vivo imaging

**DOI:** 10.1038/s41541-024-00900-5

**Published:** 2024-06-20

**Authors:** Katia Lemdani, Romain Marlin, Céline Mayet, Vladimir Perkov, Quentin Pascal, Manon Ripoll, Francis Relouzat, Nina Dhooge, Laetitia Bossevot, Nathalie Dereuddre-Bosquet, Gihad Dargazanli, Kevin Thibaut-Duprey, Jean Haensler, Catherine Chapon, Christine Prost, Roger Le Grand

**Affiliations:** 1 Université Paris-Saclay, Inserm, CEA, Center for Immunology of Viral, Auto-Immune, Hematological and Bacterial Diseases (IMVA-HB/IDMIT), Fontenay-aux-Roses & Le Kremlin-Bicêtre, France; 2 Sanofi, Marcy-L’étoile, France; 3 Integrated Drug Discovery, Sanofi, Chilly-Mazarin, France

**Keywords:** Immunology, RNA vaccines

## Abstract

The characterization of vaccine distribution to relevant tissues after in vivo administration is critical to understanding their mechanisms of action. Vaccines based on mRNA lipid nanoparticles (LNPs) are now being widely considered against infectious diseases and cancer. Here, we used in vivo imaging approaches to compare the trafficking of two LNP formulations encapsulating mRNA following intramuscular administration: DLin-MC3-DMA (MC3) and the recently developed DOG-IM4. The mRNA formulated in DOG-IM4 LNPs persisted at the injection site, whereas mRNA formulated in MC3 LNPs rapidly migrated to the draining lymph nodes. Furthermore, MC3 LNPs induced the fastest increase in blood neutrophil counts after injection and greater inflammation, as shown by IL-1RA, IL-15, CCL-1, and IL-6 concentrations in nonhuman primate sera. These observations highlight the influence of the nature of the LNP on mRNA vaccine distribution and early immune responses.

## Introduction

During the recent Covid-19 crisis, mRNA-based vaccines emerged as a highly attractive alternative to conventional vaccines due, in particular, to the safety and efficacy and rapid development cycle time^1,2^. In 2021, mRNA vaccines reached a new milestone with the marketing authorizations of the BNT162b2 and mRNA-1273 vaccines against SARS-CoV-2^3,4^.

Effective mRNA vaccines require the mRNA to be delivered and expressed in the appropriate target cells to induce antigen-specific immunity. Lipid nanoparticles (LNPs) are used to protect the mRNA against enzymatic degradation and facilitate its intracellular delivery. LNPs are typically composed of ionizable cationic lipids, phospholipids, cholesterol, and polyethylene glycol (PEG)^5,6^. The critical component of LNPs is ionizable cationic lipids, which allow mRNA complexation and enable endosomal escape of the internalized mRNA to the cytosol, where translation takes place^7,8^. In addition, ionizable cationic lipids show immunostimulatory properties and can act as adjuvants for mRNA vaccines^9^.

DLin-MC3-DMA (MC3) is an example of a well-known cationic ionizable lipid for mRNA vaccine delivery and is considered to be a gold standard for LNP manufacturing. MC3 is a component of Onpattro® (patisiran), the first Food and Drug Administration-approved RNAi drug^10,11^. DOG-IM4 is a recently developed cationic ionizable lipid for the formulation of thermostable mRNA vaccine^12^. We previously reported that unmodified mRNA encoding influenza virus hemagglutinin (mRNA-HA) is highly immunogenic in mice and can induce both B cell and Th1-biased responses when delivered in DOG-IM4 LNPs^12^ or MC3 LNPs^13^.

The characterization of vaccine biodistribution to relevant tissues is an important aspect in understanding their mechanisms of action^14^. New in vivo imaging technologies have been developed for the characterization of vaccine distribution and assessment of the dynamics of immune cells at injection sites and draining lymph nodes (dLNs)^15–20^.

Optical imaging, such as bioluminescence and fluorescence, has been used to screen LNP candidates administered via various routes and to validate the targeting of specific tissues in small animals^21,22^. Non-invasive positron emission tomography-computed tomography (PET/CT) combined with near-infrared (NIR) imaging has been used for the in vivo monitoring of mRNA vaccine trafficking to the LNs in large animal models^23^. In vivo microscopic imaging, such as fibered confocal fluorescence microscopy (FCFM), can be used for real-time, high-resolution in vivo imaging of vaccines and their interaction with local immune effectors^18,24^.

Here, we used a combination of in vivo imaging modalities for studying the migration of vaccines between the injection site and dLNs and their interactions with local immune cells. We analyzed and compared the same mRNA encapsulated in MC3 and DOG-IM4 LNPs in two complementary animal models: mice and nonhuman primates (NHPs). We also analyzed the impact of their dynamics on the early immune response.

## Results

### LNP size and mRNA encapsulation

We measured the size and mRNA entrapment of LNPs formulated with labeled mRNA-HA. Based on the results obtained using dynamic light scattering, the average size of the MC3 LNPs was 114 ± 2 nm (Mean ± SD) and that of the DOG-IM4 LNPs was 184 ± 4 nm (Mean ± SD). The polydispersity index of both LNPs was <0.1, confirming their homogeneous size distribution. The estimated encapsulation efficiency determined using the Ribogreen accessibility assay was approximately 95% (CV < 5%) for MC3 and 65% (CV < 5%) for DOG-IM4 LNPs (Table 1).

**Table 1:** Physicochemical characteristics of the LNPs

LNP	Size (nm)	PDI	Encapsulation efficiency (%)
MC3-LNP	114. 0 ± 2.0	0.08	95
DOG-IM4-LNP	184.0 ± 4.0	0.01	65

### LNPs protect mRNA and increase lymph-node targeting of the vaccine

**Fig. 1:**
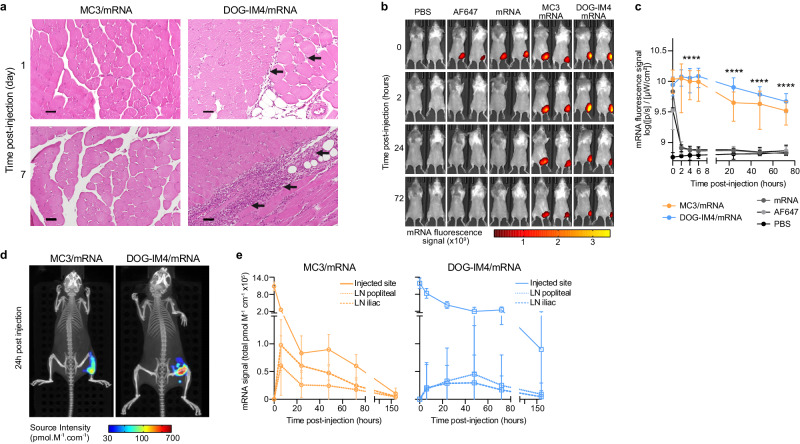
Kinetics of AF647-mRNA-HA within LNPs delivered by the i.m. route in vivo. **a** Representative images of the injection site in mice at one (top) and seven (bottom) days post i.m. injection. **b** Representative IVIS images of the AF647 fluorescent signal in mice and **c** corresponding quantification of the total radiance efficiency ((ph/s)/(µW/cm²)) in the injected area at various time points following i.m. injection of PBS (black), the AF647 probe (light gray), or 5 µg of naked AF647-mRNA (dark gray) or AF647-mRNA in MC3 (orange) or DOG-IM4 (blue) LNPs (*n* = 8/group). MC3/mRNA and DOG-IM4/mRNA groups were compared to mRNA group over time using ANOVA multiple comparisons tests (**** two-tailed *p* < 0.0001). **d** Representative recordings of 3D transillumination fluorescence tomography acquired at 24 h. **e** In vivo quantification of the biodistribution of mRNA in MC3 and DOG-IM4 LNPs at the injection site and dLNs (popliteal and iliac) over seven days (*n* = 8/group). Data are presented as mean values ± SD.

In an initial study, mice were euthanized at either day 1 (D1) or D7 following i.m administration of 5 µg mRNA within MC3 or DOG-IM4 LNPs. The injected muscle was collected, embedded in paraffin, and H&E-stained sections scored, including for neutrophil infiltration, macrophage recruitment, and skeletal muscle necrosis (Fig. 1a). In the MC3 group, we observed only slight neutrophil infiltration at D1. In the DOG-IM4 group, neutrophil infiltration was evident at D1 and completely switched to macrophage infiltration by D7. There was no impact on muscle structure, and we observed no necrosis (Table 2).

**Table 2:** Histological score in mice after MC3/mRNA or DOG-IM4/mRNA injection

	MC3		DOG-IM4	
D1	Mean	SD	Mean	SD
Neutrophilic infiltration	1.0	0.0	2.5	0.7
Macrophagic infiltration	0.0	0.0	0.0	0.0
Skeletal muscle regeneration	0.0	0.0	0.0	0.0
D7				
Neutrophilic infiltration	0.0	0.0	0.0	0.0
Macrophagic infiltration	0.0	0.0	2.0	0.8
Skeletal muscle regeneration	0.7	0.5	1.0	0.0

In a subsequent study, we tracked AF647-labeled mRNA-LNPs in mice following a single intramuscular injection (i.m., 5 µg mRNA) by whole-body fluorescence imaging. The fluorescence signal of naked mRNA (8.90 ± 0.05 log_10_ fluorescence signal, mean ± SD), as well as that of the injected AF647 probe alone (8.91 ± 0.09 log_10_ fluorescence signal, mean ± SD), was detected only at the injection site and did not last more than 120 min, indicating its rapid degradation and elimination. As compared to naked mRNA, the mRNA signal from MC3 (9.48 ± 0.16 log_10_ fluorescence signal, mean ± SD, *p* < 0.0001) and DOG-IM4 LNPs (9.65 ± 0.14 log_10_ fluorescence signal, mean ± SD, *p* < 0.0001) was detectable for up to 3 days, demonstrating the protective effect of the LNPs on the entrapped mRNA (Fig. 1b, c).

We then used tomographic fluorescence combined with trans-illumination and X-ray scanning to characterize the distribution of mRNA-LNPs in mice (Fig. 1d). Rapid migration of mRNA formulated in MC3 LNPs was observed from the injected site to the dLNs within 6 h post-injection (Fig. 1e). By contrast, the mRNA signal persisted at the injection site for up to seven days and slow trafficking to dLNs was observed for DOG-IM4 LNPs, with the signal plateauing between 24 and 48 h at lower levels than those observed in the MC3 group (Fig. 1e). At 6 h, the mRNA signal at the injection site was higher in DOG-IM4 group (*p* < 0.0001; Supplementary fig. 1a) while the mRNA signal on iliac dLNs was higher in MC3 group (*p* = 0.0266; Supplementary Fig. 1b).

### mRNA LNP vaccines are safe and have a different impact on the innate response in NHPs

**Fig. 2:**
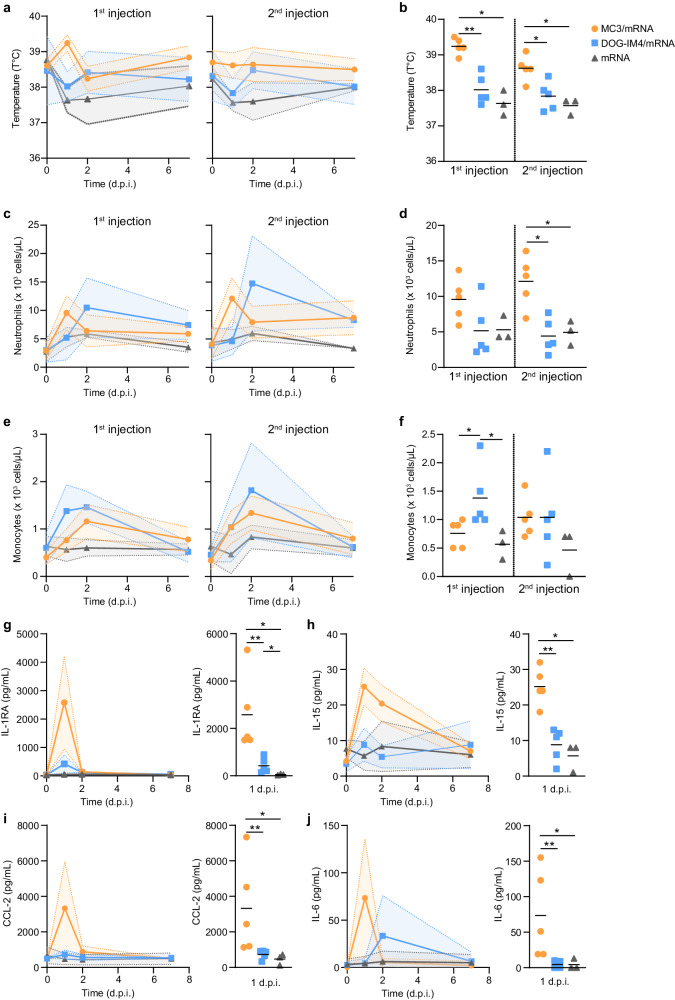
Impact of the injection of LNP/mRNA on biological parameters. Biological parameters were measured at D1, D2, and D7 post-injection (d.p.i.) with MC3/mRNA (orange), DOG-IM4/mRNA (blue), or naked mRNA (dark gray). Rectal temperature (**a**) was monitored over time after injection in each experimental group, as well as the absolute numbers of blood neutrophils (**c**) and monocytes (**e**). The lines show the mean of each experimental group, and the shaded areas represent the range. Comparison between groups of temperature (**b**), neutrophil (**d**) and monocytes (**f**) counts were performed at D1 after each injection. Serum concentrations of IL1-RA (**g**), IL-15 (**h**), CCL-2 (**i**), and IL-6 (**j**) were determined after the first injection of MC3/mRNA, DOG-IM4/mRNA, or naked mRNA. The temperature (**b**), neutrophil and monocyte counts (**d**, **f**), and serum concentrations (**g**–**j**) at D1 were compared between the groups using non-parametric Mann-Whitney and Kruskal-Wallis tests (*two-tailed *p* < 0.05, **two-tailed *p* < 0.01). Data are presented as individual and mean values ± SD (*n* = 5 for MC3/mRNA, *n* = 5 for DOG-IM4/mRNA, *n* = 3 for naked mRNA).

NHPs are considered to be particularly relevant for the preclinical evaluation of human vaccines because of the highly predictive safety, immunogenicity, and efficacy shown in these models^14^. All mRNA COVID-19 vaccines licensed for human use have been previously evaluated in NHPs^25,26^. We observed no effect on body weight following vaccine injection. The animals in the MC3/mRNA group showed an elevated rectal temperature 1 day post-first injection (mean of 39.2 °C, *p* = 0.0625) (Fig. 2a). At D1, the rectal temperature was higher for animals of the MC3/mRNA group than those of the DOG-IM4/mRNA and naked mRNA groups (*p* = 0.0079 and *p* = 0.0179, respectively; Fig. 2b). Complete blood counts showed an increase in neutrophils at D1 in the MC3/mRNA group (*p* = 0.0625) and at D2 in the DOG-IM4/mRNA group (*p* = 0.0625). Animals injected with naked mRNA showed no change (Fig. 2c). Monocyte counts also increased only in the MC3/mRNA and DOG-IM4/mRNA groups (*p* = 0.0625, Fig. 2e). After vaccination with MC3/mRNA, there was a significant increase in serum concentrations of IL1-RA (2576 ± 1638 pg/ml, mean ± SD, *p* = 0.0625), IL-15 (25.2 ± 5.2 pg/ml, *p* = 0.0625), CCL-2 (3322 ± 2633 pg/ml, *p* = 0.0625), and IL-6 (73.4 ± 62.33 pg/ml, *p* = 0.0625) at D1 (Fig. 2g–j). We also observed a peak of IL1-RA production in animals injected with DOG-IM4/mRNA (422.8 ± 320.7 pg/ml) but it was smaller than that in the animals of MC3/mRNA group (*p* = 0.008). Peak concentrations of IL-6 appeared earlier in animals of the MC3/mRNA group (D1) than in those of the DOG-IM4/mRNA group (D2) (Fig. 2j).

Overall, these results show that injection with MC3/mRNA or DOG-IM4/mRNA has a different impact on the magnitude and kinetics of early biological events, in particular, one day after injection.

### mRNA LNP localization and their interaction with APCs at the site of injection and in dLNs

**Fig. 3:**
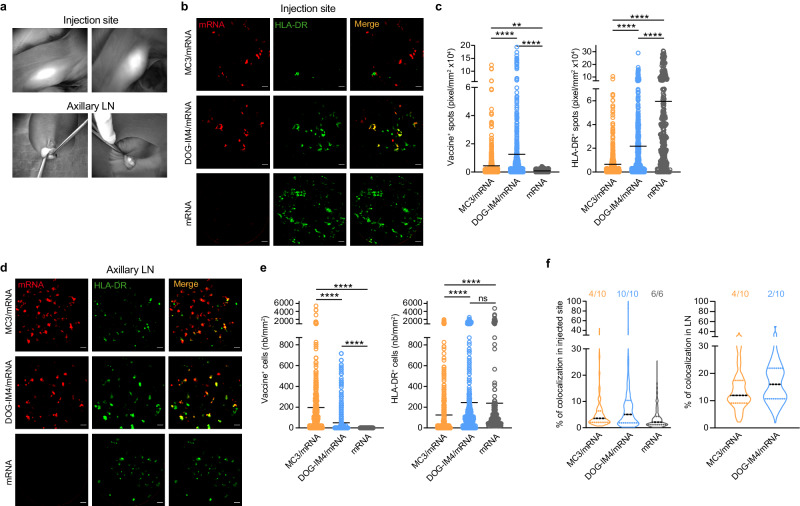
In vivo imaging of mRNA and APCs in macaques. **a** Identification of mRNA vaccine at injection sites and in dLNs by in vivo near-infrared fluorescence imaging at D1 post i.m. injection (exposure time: 100 ms). **b** Representative images captured by in vivo fibered confocal fluorescence microscopy (FCFM) at the injected sites (depth = 60 µm), with the mRNA signal in red, HLA-DR^+^ signal in green, and co-localized mRNA and HLA-DR signals in yellow. Scale bar = 20 µm. **c** Quantification of the fluorescent spots of mRNA and HLA-DR from 50 frames per imaging session randomly chosen from movies covering the zone of the injection following i.m. injection of LNP MC3/mRNA (orange), LNP DOG-IM4/mRNA (blue), or naked mRNA (dark gray). **d** Representative in vivo images obtained by the FCFM system showing mRNA^+^, HLA-DR^+^, and co-labeled mRNA^+^ and HLA-DR^+^ cells in axillary lymph nodes. **e** Quantification of the number of cells labeled for the mRNA vaccine and anti-HLA-DR from 50 frames per imaging session randomly chosen from movies covering the axillary dLN. The mean value is indicated by the horizontal bar. Experimental groups were compared using nonparametric Mann-Whitney and Kruskal-Wallis tests (**two-tailed *p* < 0.01, ****two-tailed *p* < 0.0001). **f** The percentage of co-localization was determined in mRNA positive frames and corresponded to the observation of co-labeling with HLA-DR at the injection site and in the dLNs. Images for the injection sites and dLNs were captured at one day post-injection (*n* = 10 for MC3/mRNA, *n* = 10 for DOG-IM4/mRNA, *n* = 6 for naked mRNA). Data are presented as violin plots with the quartile positions. The value shown above the violin plot indicates the number of sessions for which co-localization was observed out of all imaging sessions performed per group.

We assessed the dynamics and changes at the injection site and dLNs by first tracking mRNA-targeted antigen presenting cells (APCs) using near-infrared (NIR) in vivo imaging (Fig. 3a). This approach facilitates the combined use of in vivo confocal endomicroscopy (FCFM) to better characterize mRNA/LNP localization and HLA-DR-expressing antigen presenting cells, as we previously reported^17,18^. Naked mRNA was barely detectable at the injection site at D1 (Fig. 3b), suggesting its rapid degradation, whereas there was a significant increase in the number of HLA-DR-expressing cells (*p* < 0.0001), confirming the immune-stimulating properties of non-encapsulated mRNA (Fig. 3c).

Quantification of the mRNA and HLA-DR signals at the injection site showed a higher number of fluorescent spots associated with mRNA for the DOG-IM4 group than the other groups (*p* < 0.0001). The extent of the HLA-DR signal corresponding to the labeling of APCs in muscle was the highest in the naked mRNA group (*p* < 0.0001) (Fig. 3c).

There was no detectable signal corresponding to the fluorescent mRNA in the axillary dLNs at D1 after i.m. injection (Fig. 3d, e) for animals receiving the non-encapsulated mRNA vaccine. On the contrary, MC3 encapsulation increased the number of mRNA labeled cells in the dLNs relative to animals receiving the DOG-IM4/mRNA (196.9 ± 434.9 and 49.8 ± 133.0 cell/mm², respectively, mean ± SD, *p* < 0.0001). The number of HLA-DR-labeled cells in the dLNs was similar for the DOG-IM4/mRNA and naked mRNA groups (245.5 ± 399.3 and 238.4 ± 525.3 cell/mm², respectively) but higher than that in the MC3/mRNA group (124.0 ± 259.3 cell/mm², *p* < 0.0001).

We then analyzed the co-localization of HLA-DR labeling in mRNA-positive cells at the injection site and dLNs to determine the proportion of mRNA-targeted APCs (HLA-DR positive). The proportion of mRNA-targeted APCs for the MC3/mRNA, DOG-IM4/mRNA, and naked mRNA groups were 5.61 ± 6.49, 9.52 ± 15.38, and 3.67 ± 3.95%, respectively (mean ± SD) (Fig. 3f and Supplementary Fig. 2a). The percentage of co-localization at the injection site was higher for the DOG-IM4/mRNA than MC3/mRNA group (*p* = 0.035). In the dLNs, we observed co-localization between the mRNA vaccine and APCs for the MC3/mRNA and DOG-IM4/mRNA groups (14.35 ± 7.64 and 17.23 ± 8.75%, respectively, mean ± SD, Fig. 3f)) but not the naked mRNA group, as no mRNA-positive cells were detected (Fig. 3e and Supplementary Fig. 2b).

**Fig. 4:**
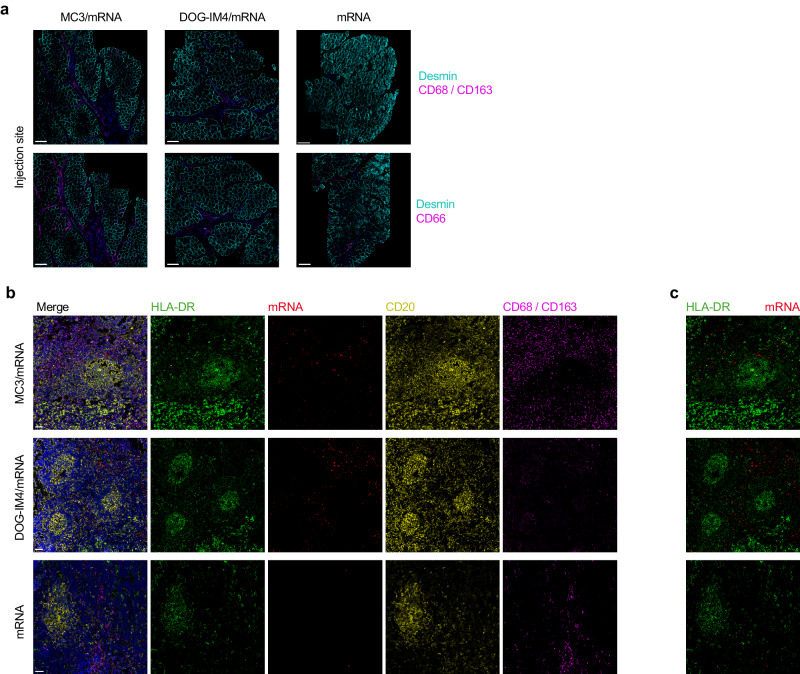
Local impact of LNP/mRNA at the injection site and in dLNs. **a** Injection sites (muscle) of NHPs at D1 post-injection with naked mRNA or mRNA in MC3 or DOG-IM4 LNPs. The sections were stained for desmin (cyan), CD68/CD163, and CD66abce (magenta). Scale bar, 200 µm. **b** mRNA-HA distribution in LNs. Representative images of mRNA localization in LNs stained for HLA-DR (green), CD20 (yellow), and CD68/CD163 (magenta) and counterstained with DAPI (Blue). Scale bar, 50 µm. **c** Magnification of areas showing co-localization of mRNA (red) and HLA-DR cells (green) in the LNs of NHPs injected with naked mRNA or mRNA in MC3 or DOG-IM4 LNPs. Images were acquired using a Leica SP8 microscope with a 40X objective. Representative images are shown (*n* = 2).

We then performed muscle and LN biopsies for two macaques per group to confirm and complete our in vivo observations. In accordance with previous published data^27^, the most abundant cells at the injection sites of LNP/mRNA-inoculated macaques were neutrophils and macrophages (Fig. 4a). In the dLNs, the mRNA of animals injected with both MC3/mRNA and DOG-IM4/mRNA was localized within the B-cell follicle area, whereas there was no detectable signal for the naked mRNA (Fig. 4b). The mRNA-positive cells were more closely associated with B cells for animals injected with MC3/mRNA than those injected with DOG-IM4. We also found macrophages to be more abundant and localized to the cortex of the dLNs of animals injected with MC3/mRNA (Fig. 4b, c).

## Discussion

We used a combination of in vivo imaging techniques to compare the dynamics and biodistribution of two LNP formulations in mice and NHPs. Replacing DLin-MC3-DMA (MC3) with the recently developed DOG-IM4 ionizable lipid in LNPs loaded with mRNA resulted in a decrease in mRNA encapsulation efficiency (from 95 to 65%) and a concomitant increase in particle size (from 114 to 184). We then compared the trafficking dynamics of the two LNP formulations following i.m. administration using a combination of high-resolution in vivo imaging techniques and immunohistochemistry to investigate early immune events. We observed distinct behavior of the two LNP/mRNA formulations at the injection sites and dLNs in both animal models. After i.m. administration, mRNA within MC3-LNPs rapidly drained to the LNs, whereas mRNA within the DOG-IM4 formulation remained longer in the injected muscle and drained to the dLNs more slowly than that in the MC3 formulation. These distinct dynamics can be explained by differences in the biophysical properties of the LNPs.

It has been shown that size affects the biodistribution of LNPs and the mechanism of cellular uptake. Particles <200 nm are believed to directly reach lymphatic drainage by convective forces, whereas larger particles may require local uptake by immune cells, especially antigen presenting cells (APCs), and subsequent transport by the APCs to the dLNs^28,29^. The smaller size of MC3 LNPs (114 nm) relative to that of DOG-IM4 LNPs (184 nm) could explain the faster drainage of MC3 LNPs to the dLNs (within 6 h) and the longer persistence of DOG-IM4 LNPs at the injection site for several days in mice. This may in turn have an impact on the recruitment of APCs and establishment of the antigen-specific immune response. Alternatively, the DOG-IM4 LNPs may be more efficiently taken up by the muscle cells or infiltrating cells than MC3 LNPs. In terms of LNP trafficking in the NHPs, both mRNA/MC3 and mRNA/DOG-IM4 could be detected in the LNs at D1, suggesting that the vaccine could freely drain to the LNs following i.m. injection. However, the mRNA signal in the dLNs at D1 was stronger in the MC3 group than the DOG-IM4 group, whereas the number of recruited APCs was higher in the muscles of NHPs that received DOG-IM4 LNPs. These observations are coherent with those made in mice and in accordance with literature, confirming the impact of LNP size on drainage to the LNs and uptake by APCs^30^. There was no fluorescent signal from the naked mRNA at the injection sites in either animal model. This can be explained by rapid mRNA degradation by RNases, which, in turn, induces chemoattraction of APCs (as observed in Fig. 2b, c) and as already reported by others^31,32^. The higher proportion of mRNA accessible to Ribogreen in the DOG-IM4 LNP (35%) versus MC3 LNP (5%) may contribute to the high APC recruitment at the injection site, as observed by in vivo FCFM (Fig. 2b, c). The distinct dynamics of MC3 and DOG-IM4 LNP drainage did not affect the development of germinal centers, as observed in NHPs that received either mRNA-LNP but not in those injected with naked mRNA. As expected, we observed increased proliferation and antigen presentation within the follicles of these germinal centers.

We also studied the effect of MC3 and DOG-IM4 LNP administration on biological parameters, such as inflammation. Local and systemic inflammation is known to occur following i.m. vaccination with MC3 formulated mRNA vaccines^27,33,34^. The inflammatory response triggered by mRNA/LNPs is typically dominated by neutrophil infiltration and the secretion of pro-inflammatory cytokine/chemokines^35^. This inflammatory response is thought to result from the combined immunostimulatory activity of mRNA and the cationic ionizable lipid component of its LNP delivery system^36–38^. In NHPs, we observed neutrophil infiltration in the muscle at 24 h post i.m. injection with both the MC3 and DOG-IM4 formulations. Neutrophil infiltration was not observed following the injection of naked mRNA on the muscle biopsy. For ethical reasons, we could only perform small biopsies on living animals. By contrast, in vivo FCFM allowed us to analyze a larger area and thus observe APC recruitment at the injection site (Fig. 3b, c). Indeed, in vivo imaging has several advantages over classical histology. Longitudinal analyses could be performed, as these techniques are less invasive, the area of analysis was larger, and it allowed coverage of the critical tissue/cells targeted by the vaccine to analyze its mechanism of action.

In NHPs, the inflammatory effect was highest in the MC3 group, based on a rapid increase in systemic neutrophil counts and body temperature, associated with a significant release of cytokines, such IL-1RA, IL-15, CCL-2, and IL-6 at D1 after injection. In comparison, the stimulation of inflammatory responses by DOG-IM4 appeared to be delayed, with a peak at D2 for IL-6 production and neutrophil dynamics. The IL-6 production following i.m. administration of LNP/mRNA has been proposed as a hallmark of the adjuvant activity mediated by the ionizable lipid component of the LNPs. This, in turn, may vary depending on the nature of this ionizable lipid^9,13^. IL1-RA is typically upregulated in response to the pro-inflammatory effect of RNA vaccines, which can be amplified by certain lipid formulations^37^.

In this report, we tried to understand the distribution of mRNA when formulated in different LNPs based on their physicochemical characteristics using in vivo imaging approaches. The immunogenicity results induced by these vaccines have been already published elsewhere^12,13^. Altogether, these works contribute to the development of these vaccine formulations.

Our study shows how the nature and physicochemical characteristics of the LNP delivery vehicle, including surface characteristics and susceptibility to release the mRNA in vivo, impact the in vivo distribution of the mRNA. The larger DOG-IM4 LNPs, compared to MC3 LNPs, persisted longer in the injected muscle, migrated less quickly to the dLNs, and induced lower systemic inflammation.

In our previous report, we studied the influenza HA-specific immune responses induced with DOG-IM4 encapsulating influenza HA mRNA^12^. This candidate conferred strong immunization properties to influenza HA mRNA in mice and macaques and a remarkable stability to the encapsulated mRNA when stored liquid in phosphate buffered saline at 4 °C. We also reported using electron microscopy analyses that DOG-IM4 LNPs encapsulating non-labeled HA mRNA show bilayer structures forming blebs emanating from electron dense bodies^12^ while MC3 LNPs show essentially solid core structures, as described by others^39^. The formation of bleb structures leads to improvement of LNP potency^40^ and may have impact on the adaptive immune response. Altogether, these works support the fact that DOG-IM4 is a good candidate for vaccine formulation.

More work is necessary to better understand the interactions between mRNA and DOG-IM4 LNPs and the correlation with in vivo mRNA distribution and the adaptive immune response. In this regard, electron microscopy work is being conducted in our group to better understand the interactions taking place between mRNA and DOG-IM4. Further mechanistic studies in mice using mRNA labels that do not interfere with mRNA translation, such as stealth labels^41^, would be useful to support the results presented in this report and to identify the relationship between mRNA uptake and cell dynamics and the quality of the adaptive immune response.

We show that it is possible to investigate early determinants of mRNA vaccine behavior and interactions with immune cells using non-invasive imaging approaches. Linking early vaccine trafficking events to downstream adaptive immune responses could potentially identify critical determinants of vaccine effectiveness. We believe that the presented vaccine monitoring approach can help to underlay mRNA vaccine mechanism of action and contributes to the development of new vaccine formulations.

## Methods

### Ethics statement and description of the animals

Female BALB/c ByJ mice (6 weeks old) were purchased from Charles River Laboratories, (Les Oncins, France). Animals were housed in the R&D animal facility of Sanofi (Campus Mérieux, 1541 Av Marcel Mérieux, 69280 Marcy l’Etoile, France), accredited by AAALAC International (Association for Assessment and Accreditation of Laboratory Animal Care) since 2011, and in accordance with European Directive 2010/63/EU. These studies were included in the project APAFIS#18358-2019010817217725v2 reviewed by the French Animal Ethics Committee number 11 and approved by the Research, Innovation and Education Ministry. Mice were anesthetized using isoflurane (ISOFLURIN 100%, Alcyon), then euthanized by a cervical dislocation. Endpoints used were general ones like 20% weight loss or decreased locomotion, but they were not reached as the protocol did not induce any noticeable adverse event.

Cynomolgus macaques (*Macaca fascicularis*), aged 35 to 42 months (13 males) and originating from Mauritian AAALAC-certified breeding centers, were used in this study. All animals were housed in the IDMIT facilities (CEA, Fontenay-aux-roses, Animal facility authorization #D92-032-02, Préfecture des Hauts de Seine, France) in compliance with European Directive 2010/63/EU, French regulations, and the Standards for the Humane Care and Use of Laboratory Animals of the Office for Laboratory Animal Welfare (OLAW, assurance number #A 5826-01, US). The protocols were approved by the institutional ethics committee “Comité d’Ethique en Expérimentation Animale du Commissariat à l’Energie Atomique et aux Énergies Alternatives” (CEtEA #44) under statement number A20-001. The study was authorized by the “Research, Innovation and Education Ministry” under registration number APAFIS#24620-2020031115133688 v1. For all handling procedures, animals were anesthetized using ketamine hydrochloride (Imalgen® 1000 5 mg/kg) associated with medetomidine hydrochloride (Domitor® 0.05 mg/kg) by i.m. route. Animals were not euthanized.

### mRNA labeling and LNP formulation

1,2-distearoyl-sn-glycero-3-phosphocholine (DSPC), PEG2000-PE, and cholesterol were obtained from Avanti Polar Lipids (Alabaster, AL, USA). MC3 was obtained from SAI Life Sciences (Hyderabad, India).

The synthetic dioleoyl lipid bearing an imidazolium head group, DOG-IM4, was synthesized as described in ref. ^12^ and obtained from SAI Life Sciences (Hyderabad, India).

High purity, custom-synthesized, non-replicative, non-modified mRNA encoding the hemagglutinin (HA) of the influenza strain A/Netherlands 2009 (H1N1) was obtained from TriLink (San Diego, CA). The Ulysis™ Alexa Fluor™ 647 Nucleic Acid Labeling Kit was obtained from Invitrogen. The fluorescent labeling of mRNA, based on the universal linkage system, was adapted according to the commercial protocol. Small scale purification of the labeled mRNA was carried out using a spin-column (Princeton separations Inc, New Jersey, USA) and larger scale purification of the nucleic acid by successive precipitation in ethanol.

LNPs were prepared as described in ref. ^12^. Briefly, an ethanol phase containing the lipids at molar ratios of 50:10:38.5:1.5 (ionizable lipid/phospholipid/cholesterol/PEGLipid), at a total concentration of 20 mg/mL, and an aqueous phase containing mRNA dissolved in 50 mM citrate buffer (pH 4.0), at 0.265 mg/mL for the preparation of DOG-IM4 or 0.305 mg/mL for the preparation of the MC3 LNPs, were prepared. The two phases were then rapidly mixed in a microfluidic mixer (NanoAssemblR, Precision Nanosystems, Vancouver, BC) at a ratio of 1:3, respectively, with a final combined flow rate of 4 mL/min. The formulations were then dialyzed (Spectrum Labs, Rancho Dominguez, CA) in 50 mM citrate buffer (pH 4.0) for at least 4 h and then in phosphate-buffered saline (pH 7.4) for 24 h. The nanoparticles were stored at 4 °C under an inert atmosphere after sterile filtration.

The size and polydispersity index of the LNPs were measured by dynamic light scattering (Zetasizer Nano ZSP, Malvern Instruments). The mRNA encapsulation efficiency was determined by mRNA accessibility to Ribogreen using the QuantiT Ribogreen RNAassay (Life Technologies, Burlington, ON).

### Administration of LNPs to mice

To evaluate mRNA delivery, mice were anesthetized using 3% isoflurane (1818290, ISOFLURIN 100%, Alcyon) and injected via the i.m. route with various LNPs (50 µl) containing 5 µg AF647 labeled mRNA-HA with 3/10 cc insulin syringes (15638708, Fisher, France), in accordance with the Sanofi internal guideline for the care and use of laboratory animals. Following LNP administration, the mice were placed on an imaging platform under continuous anesthesia (isoflurane 2%) and imaged at various time points after injection.

### Assessment of the local reaction in mice

The injected muscles of mice were collected at D1 and D7 following LNP/mRNA injection. The tissues were fixed in formalin for 24 h at RT. After fixation, the dehydrated tissues were embedded in paraffin and sections (4 μm) were stained with hematoxylin eosin and examined by a pathologist. A graduated scale from 0 to 4 based on severity for myofiber regeneration and cell infiltration within the muscle was established.

### Fluorescence imaging studies in mice

Fluorescence imaging was performed by IVIS Spectrum CT (In Vivo Imaging System, Perkin Elmer).

Epi-fluorescence imaging (2D) and 3D fluorescence imaging tomography were used in this study. For the 3D study, a trans-illumination protocol, associated with an X-ray scan of the whole body of the mouse, was used, allowing precise 3D location of the fluorescent signal. Six excitation sites were selected to trans-illuminate the mouse and thus excite the AF647-mRNA vaccine (excitation: 625 nm, emission: 650 nm).

All data were analyzed using Living Image software. The 2D fluorescence signal was quantified over a region of interest (ROI) applied to the injected area and is expressed as the total radiant efficiency (photons/s)/(μW/cm^2^). For the 3D fluorescence study, the fluorescent signal was quantified at three different ROIs consisting of the injected muscle (quadriceps), iliac LN, and popliteal LN. Quantification of the source fluorescence voxels is expressed in pmol and normalized by the subtraction of the signal background quantified in the PBS control group.

### In vivo imaging in macaques

LNPs were injected i.m. into the biceps of the animals at an interval of four weeks (D0 and D28). Then the macaques received 500 µl of LNPs containing 50 µg AF647-mRNA and 10 µg anti-HLA-DR/DP/DQ labeled with AF488. A group of three macaques, used as controls, received a solution of 500 µl PBS containing 50 µg of the labeled mRNA and 10 µg of the labeled antibody.

Monoclonal anti-human HLA-DR, DP, DQ, purchased from BDbiosciences (Cat. No 550853, cloneTu39), was covalently conjugated to the fluorophore using the Alexa Fluor™ 488 Microscale Protein Labeling Kit (A30006, Invitrogen), as described in the provided protocol. The concentrations of the protein and dye were measured using a NanoDrop 2000 spectrophotometer (Thermo Scientific^TM^). Imaging sessions were performed 24 h after each injection of LNP/mRNA + anti-HLA-DR/DP/DQ. A near-infrared (NIR) imaging system Fluobeam^TM^ 700 (*λ*_ex_: 680 nm/*λ*_em_: >700 nm; Fluoptics, Grenoble, France), already described in the study of ref. ^19^, was used to screen mRNA fluorescence at the injection site and the axillary dLNs. All images were acquired using a camera exposure time of 100 ms. Then, in vivo FCFM (Cellvizio® Dual Band, Mauna Kea Technologies, France) was performed. Briefly, an incision of the skin was made under sterile conditions in the area of interest. The UltraMiniO probe was applied directly to the muscle or the axillary dLN and moved continuously to record the distribution of the fluorescence signal from the AF647-mRNA and AF488-HLA-DR^18^.

### Quantification of fluorescent mRNA and HLA-DR and co-localization

Films were selected and exported using IC-Viewer software. Fifty images per imaging session were randomly chosen from the recorded movies. An automatic algorithm was applied to quantify the mRNA and HLA-DR fluorescence, as well as their co-localization, using ImageJ 1.6 software (National Institute of Mental Health, Bethesda, USA). Briefly, the threshold was set to reduce the background signal and the size of the selected objects ranged from 10 to 2000 pixels.

The number of fluorescent spots of mRNA (AF647-positive spot) and HLA-DR-positive cells (AF488-positive spot) was quantified. The number of spots is expressed as fluorescent pixels per mm^2^ in muscle and fluorescent cells per mm^2^ in the dLNs. For estimations of the percentage of colocalization, 50 randomized images were obtained from a pool of images showing colocalization of the mRNA and HLA-DR signals.

### Hematological parameters and inflammation

Blood cell counts and hemoglobin levels were determined from EDTA blood using a DHX800 analyzer (Beckman Coulter). Cytokines were quantified in sera using NHP Milliplex (Millipore) and a Bioplex 200 analyzer (Bio-Rad) according to the manufacturer’s instructions.

### Immunohistofluorescence of muscle biopsies and LNs

For two animals per group, muscle biopsies and axillary LNs were harvested 24 h after the second injection of LNP/mRNA. Fresh tissues were immediately fixed with 0.05 M phosphate buffer containing 0.1 M L-lysine (pH 7.4), 2 mg/ml NaIO_4_, and 4% paraformaldehyde for 6 h at 4 °C and then incubated with 30% sucrose in PBS overnight at 4 °C. The LNs were embedded in an optimal cutting temperature compound. The tissues were snap-frozen in liquid nitrogen-cooled isopentane and stored at −80 °C. The frozen tissues were cut into 10 µm-thick sections for the staining of immune cells.

Following rehydration in PBS, tissues were permeabilized for 30 min in PBS/Triton 0.3% and then blocked with PBS/BSA 10% for 30 min. For muscle staining, sections were incubated overnight at 4 °C with a mix of PBS/0.2% BSA containing anti-desmin (Clinisciences Cat. No Mob060-01; Dilution 1:500) and anti-CD66 abce (Miltenyi Cat. No 130-095-212; Dilution 1/100) antibodies. Tissue sections were then incubated with secondary antibodies Goat anti-mouse IgG1, Alexa Fluor™ 555 (Life Technologies Cat. No A-21127; Dilution 1:500) and Goat anti-mouse IgG2b, Alexa Fluor™ 594 (Life Technologies Cat. No A-21145; Dilution 1:500) for 1 h at room temperature before being immunostained for 3 h at room temperature with anti-CD68 (Biolegend Cat. No 14-0688-82; Dilution 1:200) and anti-CD163 (Biolegend Cat. No 333602; Dilution 1:71) conjugated antibodies.

For LN staining, the same protocol was used with anti-CD20 (Dako Cat. No M0755; Dilution 1:64) and anti-CD3 (BD Biosciences Cat. No 557705; Dilution 1:100) as the primary antibodies and anti-CD68 (Biolegend Cat. No 14-0688-82 Dilution 1:180), anti-CD163 (Biolegend Cat. No 333602; Dilution 1:66), anti-HLA DR, DP, DQ (BD Biosciences Cat. No 550853 Dilution 1:126) as the conjugated antibodies.

All tissue sections were stained with DAPI and mounted with Vectashield medium (H-1000, Eurobio Scientific).

We used a Leica SP8 tiling confocal microscope equipped with a 40X oil immersion objective for image acquisition and IMARIS software (Bitplane Scientific Software) for analysis.

### Data analysis

Data were collected using Excel files (Microsoft Excel 2016). Differences between unmatched groups were compared using the Mann-Whitney U test and Kruskal-Wallis test. The two-tailed Wilcoxon signed-rank test was used to compare paired conditions (clinical parameters over time in each animal group) (GraphPad Prism 8.0). ANOVA multiple comparison tests were used to compare the mean values of experimental groups together or to those of control groups (Fluorescence imaging mice data). Data are presented as mean values ± SD.

### Reporting summary

Further information on research design is available in the Nature Research Reporting Summary linked to this article.

### Supplementary information

Supplementary Information

Reporting summary

## Data Availability

The data generated during and/or analyzed during the study are available from the corresponding author upon reasonable request.

## References

[1] Pardi N, Hogan MJ, Porter FW, Weissman D (2018). mRNA vaccines—a new era in vaccinology. Nat. Rev. Drug Discov..

[2] Jackson LA (2020). An mRNA vaccine against SARS-CoV-2—preliminary report. N. Engl. J. Med..

[3] Baden LR (2021). Efficacy and Safety of the mRNA-1273 SARS-CoV-2 Vaccine. N. Engl. J. Med..

[4] Verbeke R, Lentacker I, De Smedt SC, Dewitte H (2021). The dawn of mRNA vaccines: the COVID-19 case. J. Control Release.

[5] Buschmann, M. D. et al. Nanomaterial delivery systems for mRNA Vaccines. *Vaccines* **9** (2021). 10.3390/vaccines9010065 PMC7836001 33478109

[6] Jackson NAC, Kester KE, Casimiro D, Gurunathan S, DeRosa F (2020). The promise of mRNA vaccines: a biotech and industrial perspective. NPJ Vaccines.

[7] Sabnis S (2018). A novel amino lipid series for mRNA delivery: improved endosomal escape and sustained pharmacology and safety in non-human primates. Mol. Ther..

[8] Carrasco MJ (2021). Ionization and structural properties of mRNA lipid nanoparticles influence expression in intramuscular and intravascular administration. Commun. Biol..

[9] Alameh MG (2021). Lipid nanoparticles enhance the efficacy of mRNA and protein subunit vaccines by inducing robust T follicular helper cell and humoral responses. Immunity.

[10] Rizk M, Tuzmen S (2017). Update on the clinical utility of an RNA interference-based treatment: focus on Patisiran. Pharmgenom. Pers. Med.

[11] Akinc A (2019). The Onpattro story and the clinical translation of nanomedicines containing nucleic acid-based drugs. Nat. Nanotechnol..

[12] Ripoll M (2022). An imidazole-modified lipid confers enhanced mRNA-LNP stability and strong immunization properties in mice and non-human primates. Biomaterials.

[13] Bernard MC (2023). The impact of nucleoside base modification in mRNA vaccine is influenced by the chemistry of its lipid nanoparticle delivery system. Mol. Ther. Nucleic Acids.

[14] Van Tilbeurgh M (2021). Predictive markers of immunogenicity and efficacy for human vaccines. Vaccines.

[15] McCarthy CE, White JM, Viola NT, Gibson HM (2020). In vivo Imaging technologies to monitor the immune system. Front. Immunol..

[16] Rashidian M (2015). Noninvasive imaging of immune responses. Proc. Natl Acad. Sci. USA.

[17] Todorova B (2017). Electroporation as a vaccine delivery system and a natural adjuvant to intradermal administration of plasmid DNA in macaques. Sci. Rep..

[18] Todorova B (2017). Fibered confocal fluorescence microscopy for the noninvasive imaging of langerhans cells in Macaques. Contrast Media Mol Imaging.

[19] Salabert N (2016). Intradermal injection of an anti-Langerin-HIVGag fusion vaccine targets epidermal Langerhans cells in nonhuman primates and can be tracked in vivo. Eur. J. Immunol..

[20] Romain G (2012). CD34-derived dendritic cells transfected ex vivo with HIV-Gag mRNA induce polyfunctional T-cell responses in nonhuman primates. Eur. J. Immunol..

[21] Pardi N (2015). Expression kinetics of nucleoside-modified mRNA delivered in lipid nanoparticles to mice by various routes. J. Control Release.

[22] Qiu M (2022). Lung-selective mRNA delivery of synthetic lipid nanoparticles for the treatment of pulmonary lymphangioleiomyomatosis. Proc. Natl Acad. Sci. USA.

[23] Lindsay KE (2019). Visualization of early events in mRNA vaccine delivery in non-human primates via PET-CT and near-infrared imaging. Nat. Biomed. Eng..

[24] Rosenbaum P (2018). Molecular and cellular dynamics in the skin, the lymph nodes, and the blood of the immune response to intradermal injection of modified vaccinia Ankara vaccine. Front. Immunol..

[25] Vogel AB (2021). BNT162b vaccines protect rhesus macaques from SARS-CoV-2. Nature.

[26] Corbett KS (2020). Evaluation of the mRNA-1273 vaccine against SARS-CoV-2 in nonhuman primates. N. Engl. J. Med..

[27] Liang F (2017). Efficient targeting and activation of antigen-presenting cells in vivo after modified mRNA vaccine administration in rhesus macaques. Mol. Ther..

[28] Bachmann MF, Jennings GT (2010). Vaccine delivery: a matter of size, geometry, kinetics and molecular patterns. Nat. Rev. Immunol..

[29] Thomas SN, Rohner NA, Edwards EE (2016). Implications of lymphatic transport to lymph nodes in immunity and immunotherapy. Annu. Rev. Biomed. Eng..

[30] Hassett KJ (2021). Impact of lipid nanoparticle size on mRNA vaccine immunogenicity. J. Control Release.

[31] Lu L, Li J, Moussaoui M, Boix E (2018). Immune modulation by human secreted RNases at the extracellular space. Front. Immunol..

[32] Cao Y, Gao GF (2021). mRNA vaccines: a matter of delivery. EClinicalMedicine.

[33] Bahl K (2017). Preclinical and clinical demonstration of immunogenicity by mRNA Vaccines against H10N8 and H7N9 influenza viruses. Mol. Ther..

[34] Hassett KJ (2019). Optimization of lipid nanoparticles for intramuscular administration of mRNA vaccines. Mol. Ther. Nucleic Acids.

[35] Ndeupen S (2021). The mRNA-LNP platform’s lipid nanoparticle component used in preclinical vaccine studies is highly inflammatory. iScience.

[36] Moghimi SM, Simberg D (2022). Pro-inflammatory concerns with lipid nanoparticles. Mol. Ther..

[37] Tahtinen S (2022). IL-1 and IL-1ra are key regulators of the inflammatory response to RNA vaccines. Nat. Immunol..

[38] Verbeke R, Hogan MJ, Lore K, Pardi N (2022). Innate immune mechanisms of mRNA vaccines. Immunity.

[39] Chivukula S (2021). Development of multivalent mRNA vaccine candidates for seasonal or pandemic influenza. NPJ Vaccines.

[40] Cheng MHY (2023). Induction of Bleb structures in lipid nanoparticle formulations of mRNA leads to improved transfection potency. Adv. Mater..

[41] Baladi T (2021). Stealth fluorescence labeling for live microscopy imaging of mRNA Delivery. J. Am. Chem. Soc..

