# Supplementary material for: Distinct dynamics of mRNA LNPs in mice and nonhuman primates revealed by in vivo imaging
Source: NPJ Vaccines. 2024 Jun 20;9:113. doi: 10.1038/s41541-024-00900-5 (PMC11189915; doi:10.1038/s41541-024-00900-5)
Supplement: Supplementary file 1 — Supplementary Information [file 41541_2024_900_MOESM1_ESM.pdf]

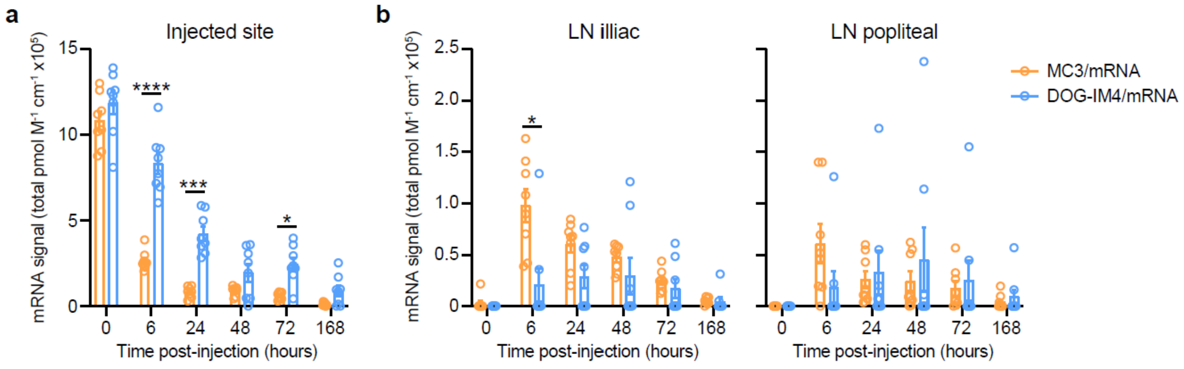

Supplementary figure 1. ***In vivo*** **imaging of mRNA and APCs in macaques.** (a-b) Quantification of the percentage of co-labelled cells with mRNA and HLA-DR from 50 frames per imaging session randomly chosen from movies covering the zone of the injection and the draining LN, following i.m. injection of LNP MC3/mRNA (orange), LNP DOG-IM4/mRNA (blue), or naked mRNA (dark grey). The number of imaging sessions with observed co-localization out of the total number of imaging sessions performed is also indicated for each group.

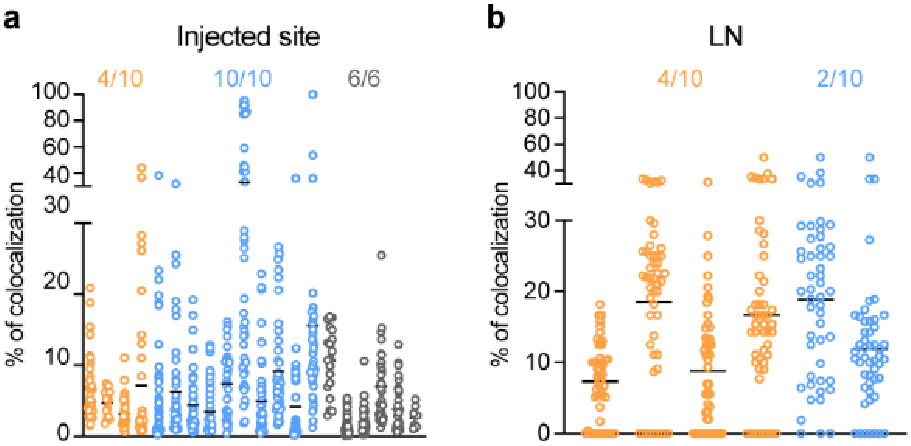

Supplementary figure 2. ***In vivo*** **imaging of mRNA and APCs in macaques.** (a-b) Quantification of the percentage of co-labelled cells with mRNA and HLA-DR from 50 frames per imaging session randomly chosen from movies covering the zone of the injection and the draining LN, following i.m. injection of LNP MC3/mRNA (orange), LNP DOG-IM4/mRNA (blue), or naked mRNA (dark grey). The number of imaging sessions with observed co-localization out of the total number of imaging sessions performed is also indicated for each group.
